# The VAPB-PTPIP51 endoplasmic reticulum-mitochondria tethering proteins are present in neuronal synapses and regulate synaptic activity

**DOI:** 10.1186/s40478-019-0688-4

**Published:** 2019-03-06

**Authors:** Patricia Gómez-Suaga, Beatriz G. Pérez-Nievas, Elizabeth B. Glennon, Dawn H. W. Lau, Sebastien Paillusson, Gábor M. Mórotz, Tito Calì, Paola Pizzo, Wendy Noble, Christopher C. J. Miller

**Affiliations:** 1 Department of Basic and Clinical Neuroscience, Maurice Wohl Clinical Neuroscience Institute, Institute of Psychiatry, Psychology and Neuroscience, King’s College London, London, SE5 9RX UK; 2 Department of Biomedical Sciences, University of Padova, Padova, Italy

**Keywords:** VAPB, PTPIP51, Endoplasmic reticulum, Mitochondria, Parkinson’s disease, Frontotemporal dementia, Amyotrophic lateral sclerosis, Synapse

## Abstract

Signaling between the endoplasmic reticulum (ER) and mitochondria regulates a number of key neuronal functions. This signaling involves close physical contacts between the two organelles that are mediated by “tethering proteins” that function to recruit regions of ER to the mitochondrial surface. The ER protein, vesicle-associated membrane protein-associated protein B (VAPB) and the mitochondrial membrane protein, protein tyrosine phosphatase interacting protein-51 (PTPIP51), interact to form one such tether. Recently, damage to ER-mitochondria signaling involving disruption of the VAPB-PTPIP51 tethers has been linked to the pathogenic process in Parkinson’s disease, fronto-temporal dementia (FTD) and related amyotrophic lateral sclerosis (ALS). Loss of neuronal synaptic function is a key feature of Parkinson’s disease and FTD/ALS but the roles that ER-mitochondria signaling and the VAPB-PTPIP51 tethers play in synaptic function are not known. Here, we demonstrate that the VAPB-PTPIP51 tethers regulate synaptic activity. VAPB and PTPIP51 localise and form contacts at synapses, and stimulating neuronal activity increases ER-mitochondria contacts and the VAPB-PTPIP51 interaction. Moreover, siRNA loss of VAPB or PTPIP51 perturbs synaptic function and dendritic spine morphology. Our results reveal a new role for the VAPB-PTPIP51 tethers in neurons and suggest that damage to ER-mitochondria signaling contributes to synaptic dysfunction in Parkinson’s disease and FTD/ALS.

**Electronic supplementary material:**

The online version of this article (10.1186/s40478-019-0688-4) contains supplementary material, which is available to authorized users.

## Introduction

Signaling between the ER and mitochondria regulates a variety of fundamental cellular processes. These include energy metabolism, Ca^2+^ homeostasis, phospholipid synthesis, mitochondrial biogenesis and trafficking, ER stress responses, autophagy and inflammation [7, 34, 37, 39]. This signaling is facilitated by close physical contacts between the two organelles such that up to approximately 20% of the mitochondrial surface is closely apposed (10–30 nm distances) to ER membranes. These regions of ER are termed mitochondria associated ER membranes (MAM) [7, 34, 37, 39].

The mechanisms by which ER membranes are recruited to the mitochondrial surface are not fully understood but it is widely agreed that the process involves “tethering proteins” which act to scaffold and anchor the two organelles in close proximity. One such tether involves an interaction between the integral ER protein, vesicle-associated membrane protein-associated protein B (VAPB) and the outer mitochondrial membrane protein, protein tyrosine phosphatase interacting protein-51 (PTPIP51) [9, 45]. The VAPB-PTPIP51 tethers are now known to facilitate inositol 1,4,5-trisphosphate (IP3) receptor mediated delivery of Ca^2+^ from ER stores to mitochondria, mitochondrial ATP production and autophagy, all of which are known to be regulated by ER-mitochondria crosstalk [9, 13, 33, 45, 46].

The pivotal roles that ER-mitochondria signaling plays in so many important physiological functions suggest that damage to this signaling will have detrimental effects on cellular homeostasis. Indeed, perturbation of ER-mitochondria contacts and signaling is associated with the major human neurodegenerative diseases Alzheimer’s disease, Parkinson’s disease and FTD/ALS [1, 23, 24, 34]. Notably, disruption to the VAPB-PTPIP51 tethers has been linked to Parkinson’s disease and FTD/ALS [9, 33, 45, 46]. FTD is the second most common form of presenile dementia after Alzheimer’s disease and is now known to be clinically, genetically and pathologically linked to ALS, the most common form of motor neuron disease [24, 28].

Loss of synaptic function is a key pathogenic feature of Parkinson’s disease and FTD/ALS. Indeed, synaptic loss underlies the cognitive and motor dysfunctions that characterise these disorders [3, 17, 43]. Both ER and mitochondria are known to be present in synaptic regions [16, 18, 31, 50]. However, any role of the VAPB-PTPIP51 tethers and ER-mitochondria signaling in synaptic function is currently unknown. Such knowledge is essential not only for comprehending the normal roles of ER-mitochondria signaling in synaptic function, but also for determining any pathological role that disruption to the VAPB-PTPIP51 tethers might play in Parkinson’s disease and FTD/ALS. Here, we show that VAPB and PTPIP51 are present and interact in synaptic regions, that their interactions are stimulated by neuronal activity, and that loss of VAPB and PTPIP51 disrupts synaptic function.

## Materials and methods

### Plasmids and siRNAs

SPLICS_s_ and SypHy-RGECO reporter plasmids were as described [6, 20]; pEGFPC1 was from Clontech. Verified non-targeting control and rat VAPB and PTPIP51 siRNAs were purchased from GE Healthcare Dharmacon (Accell range). Sequences were: VAPB A-091473-17# 5′-GUGCUGUUCUUUAUUGUUG-3′, A-091473-18# 5′- CUUAUGGAUUCAAAACUUA-3′, A-091473-19# 5′-GGUUCAGUCUAUGUUUGCU-3′, A-091473-20# 5′-GUUACAGCCUUUCGAUUAU-3′; PTPIP51 (Fam82a2) A-092062-13# 5′-CCUUUAAUGUCAUACCUUA-3′, A-092062-14# 5′-GCUUUAGCUUCAAGGAACA-3′, A-092062-15# 5′- GCUACAGCCUUGUUUGAAA-3′, A-092062-16# 5′- CUCUGGACCUUGAUAUGGA-3′.

### Antibodies and chemicals

Rabbit and rat antibodies to VAPB and PTPIP51 were as described [9]. Rabbit anti-PTPIP51 and chicken anti-MAP 2 were from Gentex. Rabbit anti-translocase of the outer mitochondrial membrane protein-20 (TOM20), mouse anti-glyceraldehyde 3-phosphate dehydrogenase (GAPDH) and goat anti-synaptophysin were from Santa Cruz Biotechnology. Mouse anti-β-Tubulin Isotype III antibody and mouse anti-β-Actin were from Sigma. Mouse anti-post synaptic density protein-95 (PSD95) was from Millipore, mouse anti-protein disulphide isomerase (PDI) was from Thermo Fisher Scientific and mouse anti-phosphorylated neurofilament heavy chain (NFH) (antibody SMI31) was from Sternberger Monoclonals Inc. Species specific goat and donkey anti-mouse, −rabbit and -chicken Igs coupled to AlexaFluor-488, − 594 or − 647 were from Invitrogen, Jackson ImmunoResearch, ThermoFisher or Abcam. FM 4–64 was from Invitrogen, DL-2-amino-5-phosphonovaleric acid (AP5) was from Cayman chemical company and 6-cyano-7-nitroquinoxaline-2,3-dione (CNQX) was from Santa Cruz.

### Hippocampal neuronal culture and transfection

Hippocampal neurons were obtained from embryonic day 18 rat embryos and cultured in Neurobasal medium containing B27 supplement, 100 IU/ml penicillin, 100 μg/ml streptomycin and 2 mM L-glutamine (Invitrogen). Neurons were cultured on poly-d-lysine-coated glass cover slips in 12-well plates and analysed at DIV20–23. For siRNA studies, neurons were untreated or treated with 1 μM of each siRNA for 72 h prior to analyses. For transfection studies, neurons were transfected at DIV5 using Lipofectamine 2000 (Invitrogen) (0.5 μg plasmid DNA, 1 μl Lipofectamine 2000 per well) according to the manufacturer’s instructions.

### Protein fractionation, SDS–PAGE and immunoblotting

Cells were harvested for sodium dodecyl sulphate–polyacrylamide gel electrophoresis (SDS-PAGE) by washing with calcium-free phosphate buffered saline (PBS) pre-warmed at 37 °C and scraping into SDS-PAGE sample buffer containing protease inhibitors (Complete Roche), 1 mM Na_3_VO_4_ and 5 mM NaF. Samples were then heated for 10 min at 100 °C, sonicated and centrifuged at 10000 g (av) for 10 min. Total, cytosolic and synaptoneurosome proteins were prepared from rat brains essentially as described [35, 47]. Protein concentrations were determined using a commercial BCA assay (Pierce). Samples were prepared for SDS-PAGE by addition of sample buffer and then resolved on 10 or 15% SDS-PAGE gels and transferred to nitrocellulose membranes (Schleicher & Schuell Bioscence) by wet electroblotting (BioRad). Membranes were blocked with Tris-HCl-buffered saline (TBS) containing 5% dried milk and 0.1% Tween-20 for 1 h at 20 °C, and then incubated with primary antibodies in blocking buffer for 16 h at 4 °C. Following washing in TBS containing 0.1% Tween-20, the blots were incubated with horseradish peroxidase conjugated secondary antibodies and developed using chemiluminescence with a Luminata Forte Western HRP substrate system according to the manufacturer’s instructions (Millipore). Chemiluminescence signals were detected using a BioRad ChemiDoc MP Imaging system.

### Immunofluorescence staining and proximity ligation assays

Neurons grown on coverslips were fixed for 15 min at 20 °C with 4% (*w*/*v*) paraformaldehyde in PBS and then permeabilized with PBS containing 0.5% Triton X-100 for 15 min. Samples were then preincubated with blocking buffer (PBS containing 10% goat or 2% donkey serum and 0.5% Triton X-100) for 1 h and incubated with primary antibodies diluted in blocking buffer for 16 h at 4 °C. Following washing in PBS containing 0.5% Triton X-100, the samples were incubated with goat/donkey anti-rabbit, mouse, rat or chicken Igs coupled to AlexaFluor − 488, − 594 or 647 in PBS for 1 h, washed in PBS and then mounted in Vectashield mounting medium (Vector Laboratories). Proximity ligation assays (PLAs) to identify the VAPB-PTPIP51 interaction were performed essentially as described previously using Duolink reagents (Sigma-Aldrich) [13]. Briefly, neurons were fixed in 4% paraformaldehyde in PBS and probed with rat anti-PTPIP51 and rabbit anti-VAPB antibodies, and signals developed using a Duolink In Situ Orange kit (Sigma-Aldrich). Following PLAs, neurons were immunolabeled for synaptophysin and PSD95.

### Microscopy

Super resolution structured illumination microscopy (SIM) was performed using Nikon Eclipse Ti-E Inverted microscopes with 100× 1.49 NA CFI objectives and equipped with Nikon N-SIM or Visitech iSIM Super Resolution Systems. Images were captured using an Andor iXon EMCCD camera and reconstructed using Nikon imaging software Elements Advanced Research with N-SIM module or Nikon deconvolution software for iSIM.

Live cell imaging was performed by time-lapse microscopy using a Nikon Eclipse Ti microscope equipped with an Intenslight C-HGFI light source, CFI Apo Lambda S 60x/1.40 objective, TiND6 PFS-S Perfect Focus Unit and EGFP, DsRed and EGFP/DsRed dual filter sets (Chroma Technology). Images were captured using an Andor Neo sCMOD camera. For dual imaging, EGFP and DsRed signals were captured simultaneously using an EGFP/DsRed dual filter set, an Andor TuCam camera adapter system equipped with an emission GFP/RFP dichroic filter set and two Andor Neo sCMOD cameras. Movements were recorded using Nikon NIS-Elements AR software at 2 or 3 s time-lapse intervals. Temperature was maintained at 37 °C using a microscope incubation chamber (Solent Scientific). During recordings, neurons were kept under constant perfusion (0.5 ml/min) with external solution using a Bio-Logic MSC200 fast perfusion system. External solution comprised 140 mM NaCl, 5 mM KCl, 5 mM NaHCO_3_, 1 mM MgCl_2_, 1.2 mM CaCl_2_, 1.2 mM Na_2_HPO_4_, 10 mM glucose in 20 mM HEPES buffer pH 7.4.

Electrical field stimulations were performed in a Chamlide EC-B18 field stimulation chamber and field stimulation (25 mA pulses of 1 ms duration) delivered by an Isolated Stimulator DS3 controlled by a Train/Delay Generator DG2A (Digitimer). Analyses involving FM 4–64 were performed essentially as described [19]. Briefly, FM 4–64 (2.5 μM) was added in external solution and loaded into neurons with electrical field stimulation (20 Hz for 60 s) which was applied with an insert adaptor in the culture plates (RC-37FS, Warner instruments). FM 4–64 dye was removed from the surface membranes by incubation in external solution containing NMDA and AMPA receptor antagonists (50 μM AP5, 10 μM CNQX) followed by incubation in Ca^2+^-free external solution. Neurons were then transferred to the microscope field stimulation chamber and analysed in time-lapse. For analyses of SPLICS_s_, field stimulations were delivered at frequency of 30 Hz for 10 s. For analyses of FM 4–64, 3 field stimulations of 60 s at 20 Hz were applied at 60 s intervals as described [19]. For analyses of SypHy-RGECO signals, 3 field stimulations of 10 s at 30 Hz were applied at 60 s intervals essentially as described [20]. VAPB-PTPIP51 PLA field stimulations were conducted in the culture plates using the insert adaptor delivering stimulations of 30 Hz for 10 s. Neurons were then fixed and processed for PLAs and immunostaining.

Confocal microscopy images were acquired using a Leica TCS-SP5 confocal microscope using a 63x HCX PL APO lambda blue CS 1.4 oil UV objective. Z-stack images were analysed and processed using Leica Applied Systems (LAS AF6000) image acquisition software.

PLA signals were quantified in close proximity (less than 1 μm) to synaptic contacts identified by synaptophysin/PSD95 apposition using NIH ImageJ in 20 μm segments of dendrites after the first dendritic branchpoint. ER-mitochondria contacts were quantified by analyses of PDI/TOM20 colocalization with Pearson’s coefficient using Nikon Imaging Software Elements AR. Dendritic spine densities were quantified using NeuronStudio software (CNIC). Active spines involving apposition of spines to synaptophysin signals were quantified using ImageJ in 20 μm segments of dendrites located after the first branch. Time-lapse movies were processed offline using the NIS-Elements AR software and ImageJ. FM 4–64 and SypHy-RGECO signals were quantified as described [19, 20].

## Results

### VAPB and PTPIP51 localise and interact at synapses

**Fig. 1:**
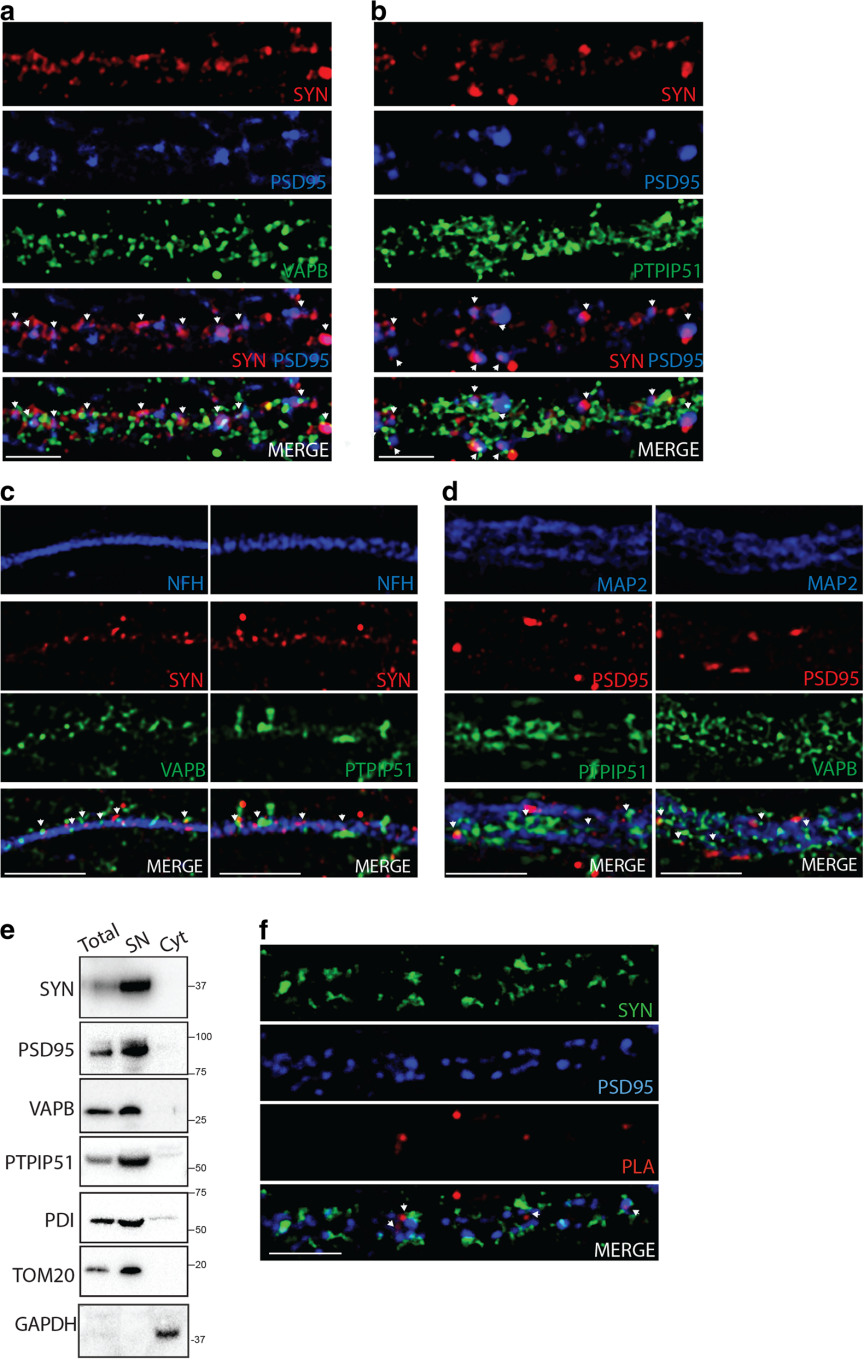
VAPB and PTPIP51 localise and interact at synapses. **a** and **b** Super-resolution SIM images of VAPB (**a**) and PTPIP51 (**b**) localisation close to synaptic pairs. Hippocampal neurons were immunolabeled for synaptophysin (SYN) and PSD95, and either VAPB or PTPIP51. SYN + PSD95 identifies synaptic contacts (arrows) via apposition of labelling. MERGE images show VAPB or PTPIP51 staining closely localised to synaptic contacts indicated by arrows shown in SYN + PSD95. **c** and **d** SIM images of VAPB and PTPIP51 localisation close to presynaptic (**c**) and postsynaptic (**d**) compartments. **c** shows presynaptic compartment identified by immunostaining for axons with phosphorylated NFH and synaptophysin. **d** shows postsynaptic compartment identified by immunostaining for dendrites with MAP2 and PSD95. Arrows in (**c**) and (**d**) MERGE indicate some VAPB and PTPIP51 labeling close to synaptophysin and PSD95. **e** VAPB and PTPIP51 are present in synaptoneurosomes. Immunoblot shows equal loading (12 μg) of total mouse brain protein, synaptoneurosome (SN) and soluble cytoplasmic protein (Cyt) fractions probed for synaptophysin (SYN), PSD95, VAPB, PTPIP51, PDI (ER marker) TOM20 (mitochondrial marker) and GAPDH (cytosolic marker). **f** VAPB-PTPIP51 PLA signals localise close to synapses in hippocampal neurons. PLAs were performed and the samples then immunostained for synaptophysin (SYN) and PSD95 to identify synapses. Arrows in MERGE indicate VAPB-PTPIP51 PLA signals close to synaptic contacts. Scale bars in a-d and f = 2 μm

We used super resolution structured illumination microscopy (SIM) to study whether VAPB and PTPIP51 localise to synaptic regions in cultured 20–23 day in vitro (DIV) rat hippocampal neurons; these neurons form mature functional synapses. We first stained neurons for synaptophysin+PSD95 and either VAPB or PTPIP51. Synaptophysin and PSD95 are pre- and postsynaptic markers and synaptic pairs can be identified by close apposition (less than 0.5 μm) of these proteins [36]. These studies revealed that both VAPB and PTPIP51 localise in close proximity to synaptic pairs (Fig. 1a, b). We then enquired whether this synaptic localisation was near presynaptic axonal boutons and/or postsynaptic dendritic spines. To identify VAPB and PTPIP51 in presynaptic regions, neurons were immunostained for phosphorylated NFH to identify axons, synaptophysin and either VAPB or PTPIP51. To identify VAPB and PTPIP51 in postsynaptic regions, neurons were immunostained for microtubule-associated protein-2 (MAP2) to identify dendrites, PSD95 and either VAPB or PTPIP51. These studies revealed that both VAPB and PTPIP51 were present in close proximity to both axonal boutons and dendritic spines (Fig. 1c, d).

**Fig. 2:**
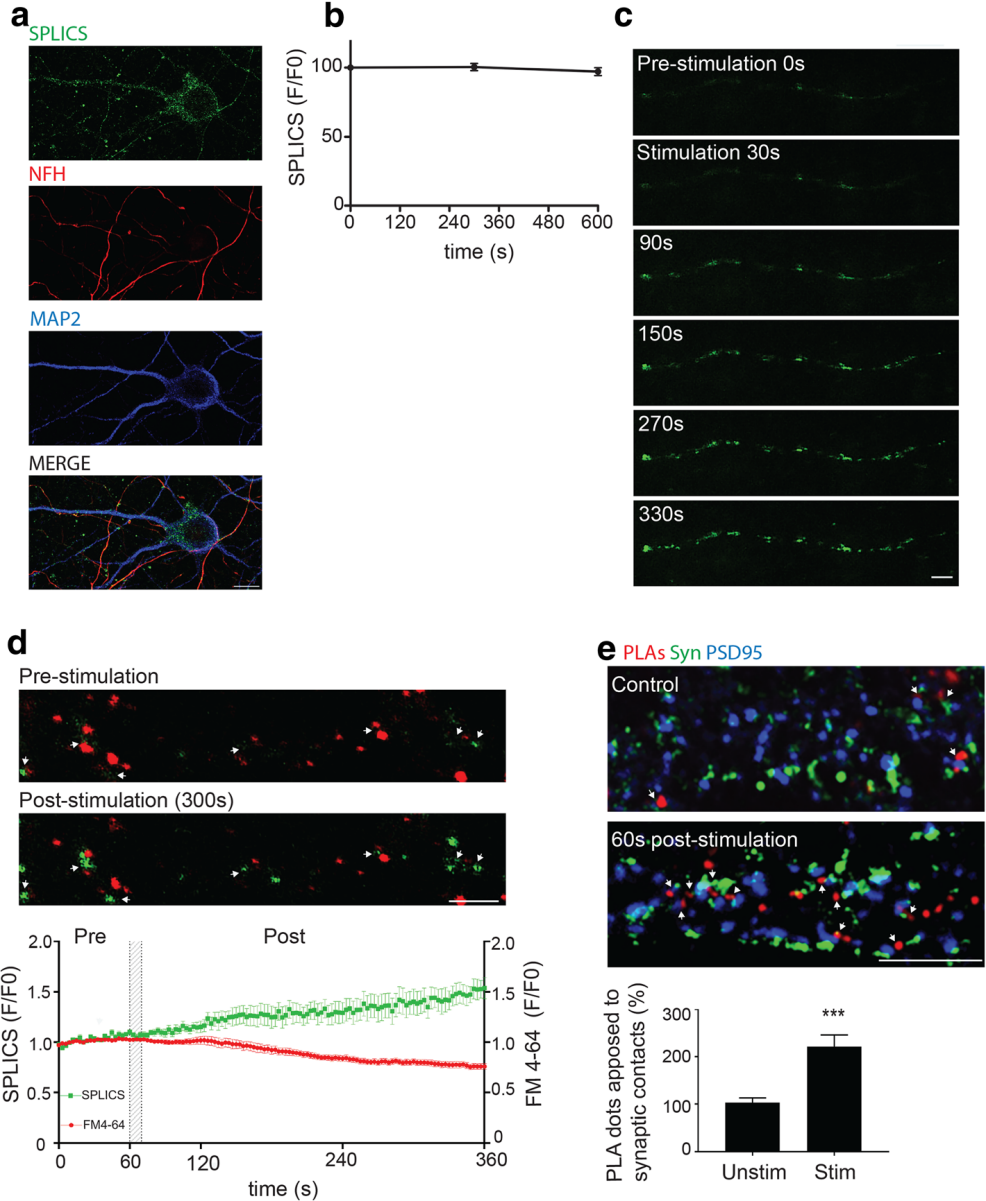
Synaptic activity regulates ER-mitochondria contacts and the VAPB-PTPIP51 interaction in hippocampal neurons. **a** Confocal z-stack of neuron transfected with SPLICS_s_ ER-mitochondria reporter plasmids and immunostained for NFH and MAP2 to show axons and dendrites; SPLICS_s_ fluorescent signal is present in cell bodies axons and dendrites. Scale bar = 10 μm. **b** SPLICS_s_ signals are stable in unstimulated neurons. SPLICS_s_ transfected neuron axons were imaged in time-lapse over a 10 min period and displayed no significant changes in SPLICS_s_ signals. Graph shows average fluorescence signal F/initial signal F0 as a %. Error bars are SEM; *N* = 14 neurons from 8 different cultures. (c and d), Electrical field stimulation of synaptic activity increases ER-mitochondria contacts including contacts close to synapses. **c** neurons transfected with SPLICS_s_ ER-mitochondria reporter plasmids were imaged in time-lapse prior to and after electrical field stimulation; image shows axon in transfected cell. **d** SPLICS_s_ transfected neurons were loaded with FM 4–64 and SPLICS_s_ (green) and FM 4–64 (red) signals imaged in time-lapse prior to and after electrical field stimulation. Arrows indicate increased SPLICS_s_ signals closely associated with active synapses identified by loss of FM 4–64 signal. Graph shows changes in fluorescence signal (fluorescence at each time-point (F)/average of all pre-stimulation frames (F0)) from 7 synapses. Error bars are SEM. Analyses of SPLICS_s_ signal reveals no changes prior to stimulation in agreement with data shown in (**b**) above but significant increases following stimulation (time 30–60 s; prior to stimulation v time 300–360; post stimulation *p* ≤ 0.0001, one way ANOVA with Tukey’s post hoc test). **e** Representative SIM image of VAPB-PTPIP51 PLA signals in synaptic regions of unstimulated neurons and in neurons after (60 s) electrical field stimulation. VAPB-PTPIP51 PLAs (red) were performed and neurons then immunostained for synaptophysin (green) and PSD95 (blue). Arrows indicate PLA signals close (less than 1 μm) to synapses as identified by apposition of synaptophysin and PSD95 signals. Bar chart shows normalised VAPB-PTPIP51 PLA signals (%) close to synapses in unstimulated neurons and in neurons after electrical field stimulation. Data were analysed by Student’s t test. *N* = 36 neurons unstimulation and 34 neurons post stimulation from 3 independent experiments. Error bars are SEM; ***p ≤ 0.001. Scale bars in **c**, **d** and **e** = 2 μm

To complement these studies, we prepared biochemical fractions of synaptoneurosomes from rat brain and probed these on immunoblots for VAPB and PTPIP51, synaptophysin and PSD95 as synaptic markers, and PDI and TOM20 as ER and mitochondrial markers. Such synaptoneurosome preparations have been extensively characterised and contain the presynaptic synaptosome with attached postsynaptic neurosome [35, 47]. For these immunoblots we also loaded equal amounts of total mouse brain and cytosolic (soluble) proteins for comparison. These studies revealed that VAPB and PTPIP51 were present in synaptoneurosomes (Fig. 1e). Finally, we performed proximity ligation assays (PLAs) to determine whether VAPB and PTPIP51 are closely associated with each other in synaptic regions in the cultured hippocampal neurons. The distances required for PLA signals are similar to those detected by resonance energy transfer between fluorophores (i.e. approximately 10 nm) and so PLAs are suitable for quantifying ER-mitochondria contacts of 10–30 nm [42]. Indeed, PLAs including ones for VAPB and PTPIP51 have already been used to quantify ER-mitochondria contacts [9, 13, 16, 33, 46]. Synapses were identified by close apposition of synaptophysin and PSD95 immunofluorescent signals. These studies revealed that VAPB-PTPIP51 PLA signals were closely associated with synapses (less than 1 μm distance) (Fig. 1f) (and see Fig. 2c). Thus, VAPB and PTPIP51 localise and interact in synaptic regions.

### Synaptic activity stimulates ER-mitochondria contacts and the VAPB-PTPIP51 interaction

The localisation and interaction of VAPB and PTPIP51 in synaptic regions suggest they play a role in synaptic function. We therefore monitored how synaptic activity affects ER-mitochondria contacts and the VAPB-PTPIP51 interaction. To do so we first utilised split green fluorescent protein (GFP) ER-mitochondria contact reporter (SPLICS) plasmids that comprise outer mitochondrial membrane and ER targeting sequences fused respectively to the GFP1–10 and β-strand 11 of the superfolder GFP variant [6]. This SPLICS_s_ contact sensor only fluoresces when the two organelles are brought into close (approximately 10 nm) proximity so as to restore GFP functional domains [6]. SPLICS_s_ transfected hippocampal neurons displayed fluorescence signals in cell bodies, axons and dendrites demonstrating close ER-mitochondria contacts in the cells (Fig. 2a). The intensities of these SPLICS_s_ signals displayed no noticeable changes over extended monitoring periods (10 min) which indicates that ER-mitochondria contacts are stable in the neurons (Fig. 2b). This observation is consistent with previous studies of SPLICS_s_ transfected cells which show stable signals over many hours [6]. However, induction of synaptic activity by electrical field stimulation [19, 20] markedly increased the SPLICS_s_ signals in cell bodies and processes (Fig. 2c and Additional file 1: Movie 1).

To determine whether any of these increased SPLICS_s_ fluorescent signals were associated with active synapses, we loaded the neurons with the red fluorescent synaptic vesicle recycling dye FM 4–64 and monitored both FM 4–64 and SPLICS_s_ fluorescent signals prior to and following electrical field stimulation. During loading, FM 4–64 is taken into synaptic vesicles as they form via endocytosis; however, during synaptic activity FM 4–64 fluorescent signals are reduced as vesicles are released from active synapses [19]. Electrical field stimulation induced selective decreases in FM 4–64 signals of approximately 30%; these reductions are in line with those reported by others following similar treatments [27, 30, 48] (Fig. 2d). These decreases were accompanied by increased SPLICS_s_ fluorescence indicating increased ER-mitochondria contacts (Fig. 2d). Notably, many of these increased SPLICS_s_ signals localised close to active synapses as identified by reduction of FM 4–64 signals (Fig. 2d and Additional file 2: Movie 2).

We also enquired whether the increases in ER-mitochondria contacts induced by synaptic activity in the hippocampal neurons were linked to changes in the VAPB-PTPIP51 interaction. To do so, we performed VAPB-PTPIP51 PLAs on unstimulated neurons and on neurons following electrical field stimulation, and monitored whether any changes in PLA signals were close to synapses. Synapses were identified by apposition of synaptophysin and PSD95 immunofluorescent signals. Quantification of these PLA signals were in the same regions of unstimulated and stimulated neurons (20 μm segments after the first dendritic branchpoint). Electrical field stimulation increased the numbers of VAPB-PTPIP51 PLA signals and this included signals that were close (less than 1 μm distance) to synapses (Fig. 2e).

### Loss of VAPB and PTPIP51 reduce dendritic spine numbers and synaptic activity

**Fig. 3:**
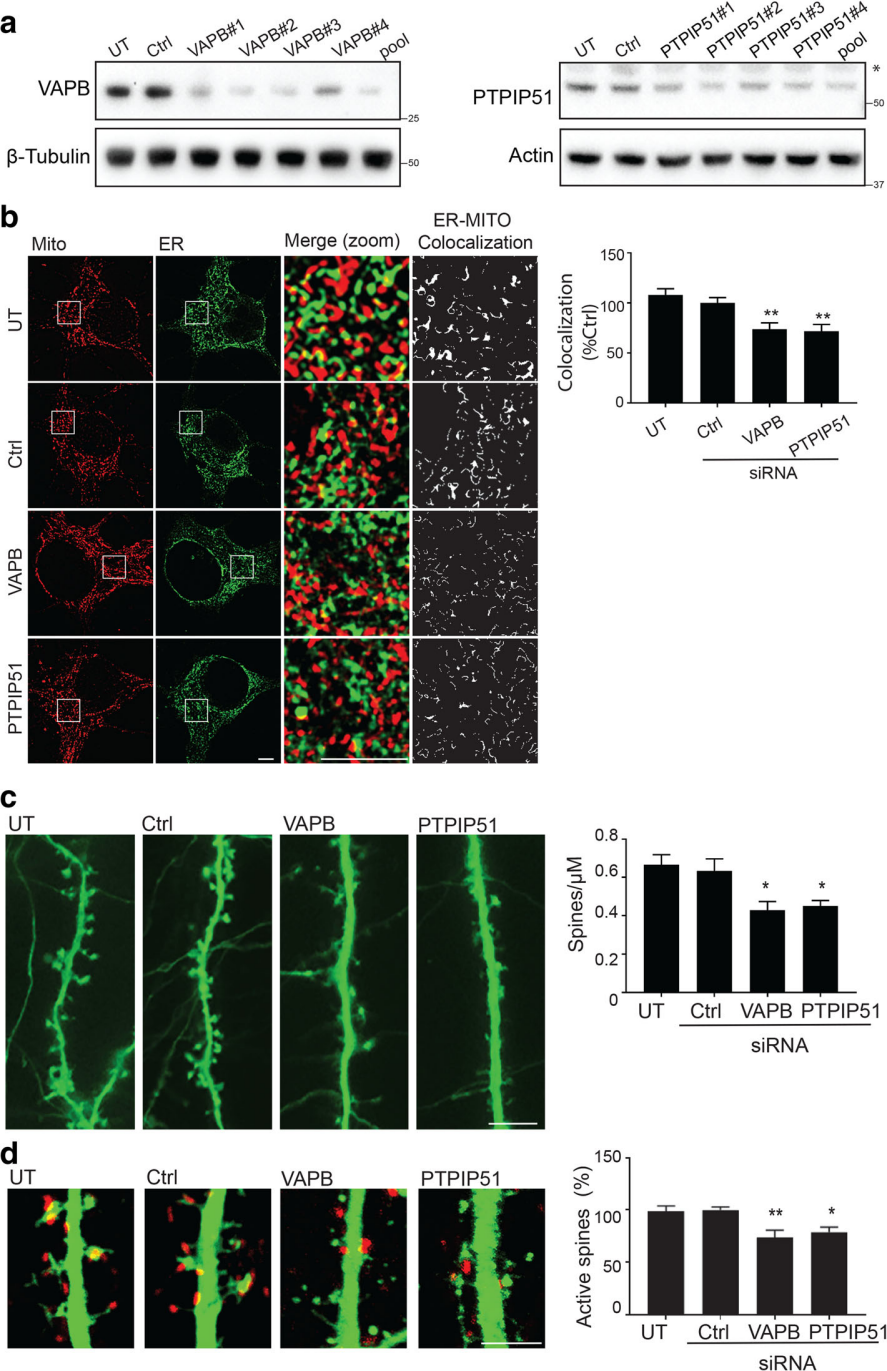
Loss of VAPB and PTPIP51 disrupts ER-mitochondria contacts and reduces dendritic spine and active spine numbers in hippocampal neurons. **a** Immunoblots showing VAPB and PTPIP51 levels in hippocampal neurons either untreated (UT) or treated with control (Ctrl), 4 different VAPB/PTPIP51 siRNAs or a pool of these siRNAs. PTPIP51 migrates at approximately 61 kD in agreement with previous studies [9, 45]. The weakly staining upper minor species (*) on the PTPIP51 immunoblot does not display any consistent changes in response to the PTPIP51 siRNAs and so we believe it to be non-specific protein. We did not detect any other PTPIP51 species in the neurons. **b** Super resolution SIM images of neurons either untreated or treated with control, VAPB or PTPIP51 pooled siRNAs, and then immunostained for ER and mitochondria with PDI and TOM20 antibodies. Zooms are of boxed regions with merge and co-localisation of signals. Scale bars = 5 μm. Bar chart shows ER–mitochondria co-localisation normalized to control siRNA in the different samples. Data were analysed by one-way ANOVA with Tukey’s post hoc test. *N* = 19 neurons UT, *N* = 42 neurons Ctrl siRNA, *N* = 42 neurons VAPB siRNA, *N* = 40 neurons PTPIP51 siRNA from 3 independent experiments; error bars are SEM, **p ≤ 0.01. **c** Representative images of dendritic spines in EGFP transfected neurons either untreated or treated with control, VAPB or PTPIP51 siRNAs. Bar chart shows spine densities (spines/μm). Data were analysed by one-way ANOVA with Tukey’s post hoc test. *N* = 14 neurons UT, *N* = 21 neurons Ctrl siRNA, *N* = 17 neurons VAPB siRNA, *N* = 21 neurons PTPIP51 siRNA from 3 independent experiments; error bars are SEM, *p ≤ 0.05. Scale bar = 5 μm. **d** Representative images of active dendritic spines in EGFP transfected neurons either untreated or treated with control, VAPB or PTPIP51 siRNAs. Active spines were identified by immunostaining for the presynaptic marker synaptophysin (red) and monitoring spine and synaptophysin apposition. Bar chart shows % of active spines normalised to control siRNA treatment. Data were analysed by one-way ANOVA with Tukey’s post hoc test. *N* = 19 neurons UT, *N* = 20 neurons Ctrl siRNA, *N* = 22 neurons VAPB siRNA, *N* = 27 neurons PTPIP51 siRNA from 3 independent experiments; error bars are SEM, *p ≤ 0.05, **p ≤ 0.01. Scale bar = 3 μm

The above findings suggest that the VAPB-PTPIP51 tethers play a role in synaptic function. To test this possibility further, we first enquired how siRNA loss of VAPB or PTPIP51 affects dendritic spine numbers in the hippocampal neurons since spine numbers are closely linked with synapse function [21]. siRNAs for VAPB and PTPIP51 have been characterised previously [9, 13, 45] and we confirmed that these siRNAs reduced VAPB and PTPIP51 protein levels in the rat neurons (Fig. 3a). Individual VAPB/PTPIP51 siRNAs were all effective as were the mixed “pools” of these siRNAs. The pooled siRNAs led to approximate 82% (± 9.1%) and 66% (±2.9%) reductions in VAPB and PTPIP51 levels respectively, and were used in all later experiments. We also confirmed that as in other cell types, this loss of VAPB and PTPIP51 reduced ER-mitochondria contacts in the neurons by performing super resolution SIM on untreated, control, VAPB and PTPIP51 siRNA treated neurons that were immunostained for PDI and TOM20 to label ER and mitochondria respectively. As predicted, loss of VAPB or PTPIP51 both reduced ER-mitochondria contacts in the neurons (Fig. 3b).

To determine how siRNA loss of VAPB or PTPIP51 affects dendritic spine numbers, we transfected neurons with EGFP to reveal neuronal morphology and identify dendrites; such approaches have been used in many other studies e.g. [8, 11, 49]. We then quantified spine numbers in the same dendritic regions of the different treated neurons (20 μm segments after the first branchpoint). Loss of VAPB or PTPIP51 reduced spine numbers (Fig. 3c). We also determined how loss of VAPB/PTPIP51 affected spines that are part of active synaptic pairs by immunostaining the EGFP transfected neurons for synaptophysin. Apposition of spines with synaptophysin immunolabelling can be used to identify active spines [49]. Loss of VAPB or PTPIP51 also reduced active spine numbers (Fig. 3d).

**Fig. 4:**
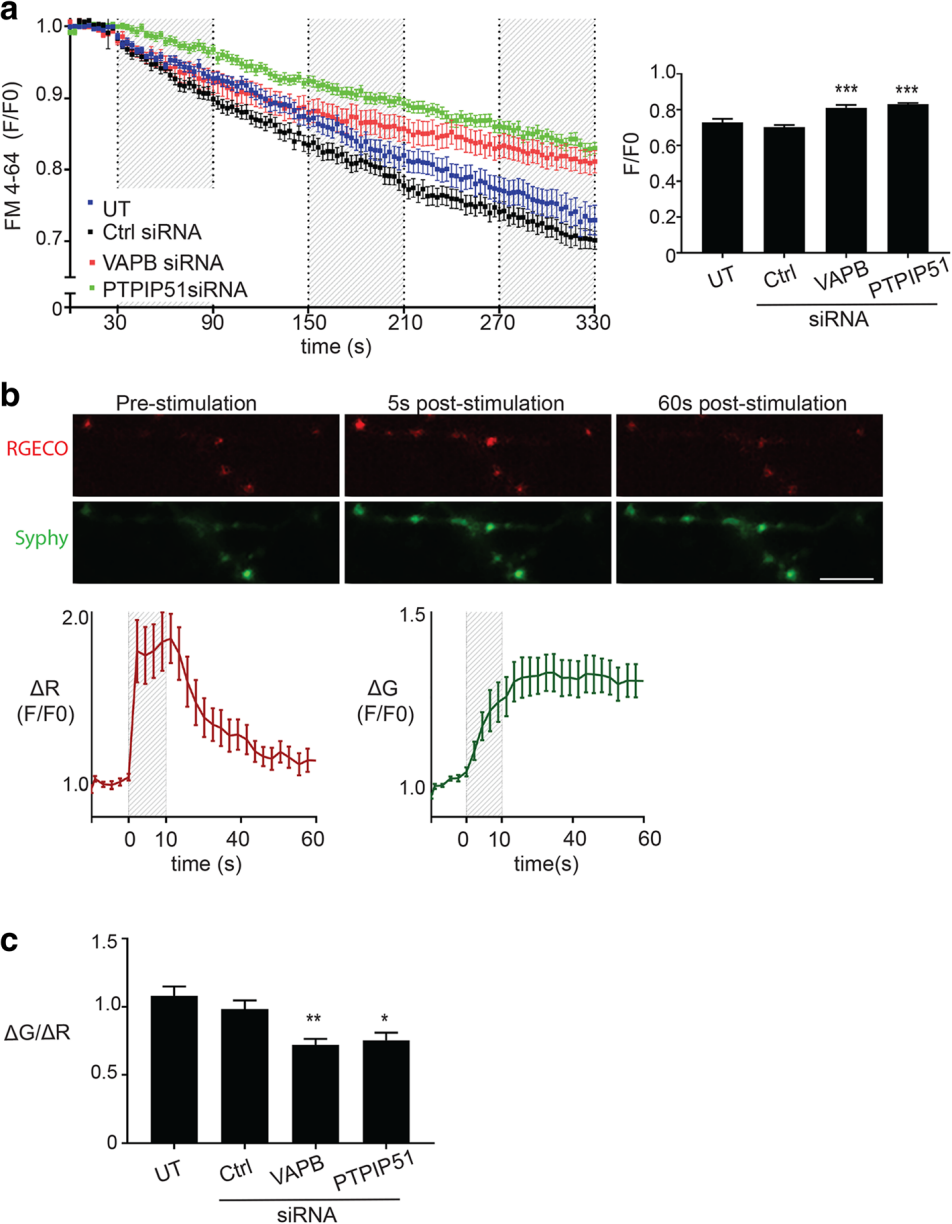
Loss of VAPB or PTPIP51 disrupts synaptic activity. **a** Kinetics of FM 4–64 release from synaptic boutons in hippocampal neurons either untreated (UT) or treated with control (Ctrl), VAPB or PTPIP51 siRNAs. Neurons were loaded with FM 4–64 and synaptic activity induced by electrical field stimulation. Periods of electrical field stimulation are indicated by shaded regions. FM 4–64 signals were determined from images acquired by time-lapse microscopy. F/F0 represents the ratio of the FM 4–64 fluorescent signals at each time point to signals at time 0. Error bars are mean ± SEM. Bar chart shows F/F0 FM 4–64 fluorescent signals at time 330 s. Data were analysed by one-way ANOVA with Tukey’s post hoc test. *N* = 24 boutons UT, N = 40 boutons Ctrl siRNA, N = 24 boutons VAPB siRNA, *N* = 35 boutons PTPIP51 siRNA from 3 independent experiments. Error bars are SEM, ***p ≤ 0.001. **b** Representative images of transfected SypHy-RGECO axonal signals in neurons prior to and following electrical field stimulation to induce synaptic activity. Scale bar = 1.5 μm. Graphs show changes in SypHy (ΔG) and RGECO (ΔR) fluorescent signals; shading shows time of electrical field stimulation. *N* = 18 synapses; error bars are SEM. **c** SypHy-RGECO reporter reveals reduced synaptic responses to electrical field stimulation in VAPB and PTPIP51 siRNA treated hippocampal neurons. Bar-graph shows the ratio between SypHy (ΔG) and RGECO (ΔR) fluorescence signals in response to electrical field stimulation. Data were analysed by one-way ANOVA and Tukey’s post hoc test. *N* = 39 synapses UT, *N* = 48 synapses Ctrl siRNA, *N* = 59 synapses VAPB siRNA, *N* = 41 synapses PTPIP51 siRNA from 3 to 4 independent experiments. Error bars are SEM; ***p ≤ 0.001

Since morphological changes in synapses are linked to synaptic function, we next studied how loss of VAPB or PTPIP51 affects synaptic activity. We first monitored release of pre-loaded FM 4–64 dye following electrical field stimulation in control, VAPB and PTPIP51 siRNA knockdown neurons. As shown above (Fig. 2d) and by others [19], electrical field stimulation induced release of FM 4–64 from synapses with concomitant decreases in dye signals. However, siRNA knockdown of VAPB or PTPIP51 inhibited this loss of FM 4–64 signal (Fig. 4a).

We also utilised a genetic indicator reporter plasmid that permits imaging of both presynaptic Ca^2+^ influx and vesicle exocytosis. This reporter (SypHy-RGECO) involves fusion of the synaptic vesicle protein synaptophysin to both a red shifted Ca^2+^ indicator (RGECO1) and a GFP-based pH sensor (pHluorin). This enables optical correlates of Ca^2+^ and pH changes to be simultaneously monitored in synaptic vesicles [20]. Due to the fixed stoichiometry of the two probes, the ratio of the two responses can be used to provide an optical correlate of the Ca^2+^ dependence of vesicle release and so provide a measure of presynaptic activity [20]. Hence, the correct way of reporting data obtained from the SypHy-RGECO indicator is to display the ratio of SypHy and RGECO signals (SypHy/RGECO) [20]. SypHy-RGECO transfected neurons displayed punctate fluorescent signals in both channels and electrical field stimulation induced increases in these signals as described by others [20] (Fig. 4b). However, compared to control cells, the ratio of SypHy/RGECO amplitudes of these signals were reduced in both VAPB and PTPIP51 siRNA treated cells consistent with diminished presynaptic activity (Fig. 4c). Thus, loss of VAPB and PTPIP51 reduces dendritic spine and active spine numbers, and decreases synaptic activity following electrical field stimulation.

## Discussion

Loss of synapses and synaptic dysfunction are principal features of the major human neurodegenerative diseases Alzheimer’s disease, Parkinson’s disease and FTD/ALS [3, 17, 43]. Damage to ER-mitochondria contacts and signaling is increasingly linked to these diseases and for Parkinson’s disease and FTD/ALS this includes disruption to the VAPB-PTPIP51 ER-mitochondria tethering proteins [1, 23, 33, 34, 45, 46]. However, whilst ER and mitochondria are both present in synaptic regions [16, 18, 31, 50] any role that the VAPB-PTPIP51 tethers play in synaptic function is not currently known. Such knowledge is essential for determining whether disruption to ER-mitochondria signaling and the VAPB-PTPIP51 tethers contributes to synaptic dysfunction in Parkinson’s disease and FTD/ALS.

Here we show that the VAPB-PTPIP51 ER-mitochondria tethers are present and interact at synapses. We also demonstrate that stimulating synaptic activity increases ER-mitochondria contacts in synaptic regions and that this involves increased interactions between VAPB and PTPIP51. Finally, we show that siRNA loss of VAPB or PTPIP51 to reduce ER-mitochondria contacts inhibits synaptic activity including alterations to synaptic vesicle release and dendritic spine numbers. Together, our results demonstrate that ER-mitochondria contacts mediated by the VAPB-PTPIP51 tethers regulate synaptic function.

To determine how VAPB and PTPIP51 siRNA loss affects presynaptic function, we utilised two experimental approaches. The first involved the synaptic vesicle recycling dye FM 4–64 which is loaded into synaptic vesicles as they form via endocytosis and then released following induction of synaptic activity [19]. Loss of VAPB and PTPIP51 both reduced FM 4–64 release consistent with inhibition of presynaptic activity. The initial loading of FM 4–64 requires stimulation of synaptic activity and so it would be interesting in future studies to determine whether loss of VAPB and PTPIP51 affects this process. Such studies would assist in determining whether the VAPB-PTPIP51 tethers affect synaptic vesicle endocytosis and recycling.

The second approach utilised a genetic indicator SypHy-RGECO that involves fusion of the synaptic vesicle protein synaptophysin to both a red shifted Ca^2+^ indicator (R-GECO1) and a GFP-based pH sensor (pHluorin). Synaptic activity induces increased Ca^2+^ levels and also changes in pH which are the result of release of neurotransmitter from the acidic synaptic vesicle into the more basic synaptic cleft. Since the pHluorin sensor is more active in basic conditions, stimulation of synaptic activity generates increases in both R-GECO1 and pHluorin signals. The SypHy-RGECO indicator therefore enables optical correlates of Ca^2+^ and pH changes to be simultaneously monitored in synaptic vesicles. As was the case with FM 4–64 experiments, loss of VAPB and PTPIP51 both inhibited presynaptic activity in assays involving SypHy-RGECO.

As detailed above, following their release, synaptic vesicles are endocytosed for re-cycling and this involves their re-acidification. Following stimulation of synaptic activity, pHluorin signals initially increase with synaptic vesicle release but then decrease as the vesicles are endocytosed. Over the times analysed in our experiments, we observed the expected increase in pHluorin signals following induction of synaptic activity but no marked decreases. However, the times taken for these decreases are variable and dependent firstly upon endocytosis rates and then the times taken for re-acidification of vesicles by vATPase proton pumps. The kinetics are also dependent upon experimental conditions such as the strength of electrical stimulation used to induce synaptic activity [38] and the type and strength of buffer used to bathe the neurons; stronger buffers require longer to acidify [2]. Finally, different indicator plasmids (e.g. fusion of pHluorin to synaptophysin, synaptobrevin and VGLUT1) can provide differences in rates of endocytosis. Interestingly, like us others have shown that the SypHy-RGECO indicator plasmid we use generates relatively stable high signals following electrical field stimulation [20]. Future studies that involve analyses of SypHy-RGECO signals at later time points will help determine how the SypHy-RGECO indicator responds to vesicle recycling.

Aside from these presynaptic effects, we also found that siRNA loss of VAPB and PTPIP51 decreased total dendritic spine numbers and also the numbers of active spines as determined by their apposition to presynaptic synaptophysin. The VAPB-PTPIP51 ER-mitochondria tethers therefore have roles in both pre- and postsynaptic function. Others have recently shown that ER-mitochondria signaling can affect postsynaptic function although whether this involved the VAPB-PTPIP51 tethers was not reported [18]. Since synapse function is intimately related to both pre- and postsynaptic changes, these reductions in dendritic spine number induced by loss of VAPB and PTPIP51 may therefore influence some of the presynaptic changes we observe.

The precise mechanisms by which the VAPB-PTPIP51 ER-mitochondria tethers affect synaptic function are not fully clear. However, dynamic changes in Ca^2+^ signaling are fundamental to synaptic transmission. Presynaptic Ca^2+^ levels regulate neurotransmitter release and dendritic Ca^2+^ alterations control synaptic plasticity [10, 40, 41, 44]. Synaptic transmission is also metabolically expensive and ATP production to drive this transmission is associated with mitochondrial Ca^2+^ levels since several dehydrogenases in the tricarboxylic acid cycle are Ca^2+^ regulated [12, 14, 15, 25, 34]. A primary function of ER-mitochondria contacts including those mediated by the VAPB-PTPIP51 tethering proteins is to facilitate delivery of Ca^2+^ to mitochondria from ER stores [7, 9, 12, 15, 34, 45]. Indeed, the VAPB-PTPIP51 tethers have been linked to mitochondrial ATP production [13, 33, 46]. Our findings that the VAPB-PTPIP51 tethers regulate synaptic function are therefore consistent with the known roles of these proteins in Ca^2+^ homeostasis and the generation of mitochondrial ATP.

Aside from these roles, ER-mitochondria contacts also control a number of other fundamental cellular functions. For example, the contacts regulate lipid metabolism since the synthesis of some phospholipids involves precursor exchange between the two organelles and the tight contacts facilitate this exchange [7, 12, 34]. ER-mitochondria signaling involving the VAPB-PTPIP51 tethers also affects autophagosome formation [13]. Changes in lipid metabolism and autophagy are both known to influence synaptic function [26, 32]. Thus, the VAPB-PTPIP51 tethers may also modulate synaptic function via their roles in these other processes.

Finally, PTPIP51 has been associated with changes in the activities of a number of signaling molecules and in particular, mitogen-activated protein kinase (MAP kinase) [4]. MAP kinase plays major roles in synaptic function [29]. In addition, and based upon its expression in different brain regions, PTPIP51 has been linked to learning and memory [5]. It will be interesting to determine whether the role of PTPIP51 and VAPB in regulating ER-mitochondria Ca^2+^ leads to downstream changes in MAP kinase and other signaling molecules.

As detailed above, damage to ER-mitochondria signaling has been associated with Alzheimer’s disease, Parkinson’s disease and FTD/ALS and for Parkinson’s disease and FTD/ALS this can involves disruption of the VAPB-PTPIP51 tethers [1, 23, 24, 33, 34, 45, 46]. Synaptic damage is a key feature of all these diseases but the mechanisms underlying this damage are not fully understood. Our findings that the VAPB-PTPIP51 tethers regulate synaptic activity therefore provide a novel route linking neurodegenerative disease insults with synaptic dysfunction. Future studies to determine whether such insults affect ER-mitochondria contacts and the VAPB-PTPIP51 tethers in synaptic regions would provide further insight into this topic. In addition, it will also be informative to determine whether correction of damaged VAPB-PTPIP51 tethers rescues synaptic damage. Interestingly, increasing ER-mitochondria contacts via overexpression of VAPB rescues α-synuclein induced damage to mitochondrial Ca^2+^ levels [33]. Also, exogenous viral delivery of VAPB is protective in ALS mutant superoxide dismutase-1 transgenic mice [22]. Thus, the findings we report here pave the way for future studies that address whether synaptic damage in neurodegenerative diseases is linked to changes in VAPB-PTPIP51 interactions and ER-mitochondria signaling.

## Conclusions

Damage to ER-mitochondria signaling is now known to contribute to Alzheimer’s disease, Parkinson’s disease and FTD/ALS. For Parkinson’s disease and FTD/ALS, this damage has been shown to involve disruption to the ER-mitochondria tethering proteins VAPB and PTPIP51 which function to recruit regions of ER to the mitochondrial surface. Loss of synaptic function is a key pathogenic feature of Parkinson’s disease and FTD/ALS. Both ER and mitochondria are present in synaptic regions but whether ER-mitochondria signaling involving the VAPB-PTPIP51 tethers contributes to synaptic function is not known. Here we show that the VAPB-PTPIP51 tethering proteins are present and interact in synaptic regions and that loss of VAPB and PTPIP51 perturbs synaptic activity. Thus, damage to the VAPB-PTPIP51 tethers may contribute to synaptic dysfunction in Parkinson’s disease and FTD/ALS.

## Additional files

Additional file 1: **Movie 1.** Electrical field stimulation of synaptic activity increases ER-mitochondria contacts. Movie shows SPLICS_s_ fluorescent signals in hippocampal neurons prior to and after stimulation. Time in seconds (s) are shown. CB indicates cell body. Electrical field stimulation was applied at 30s. (MOV 10446 kb)

Additional file 2: **Movie 2.** Electrical field stimulation of synaptic activity increases ER-mitochondria contacts including contacts close to synapses. Movie shows SPLICS_s_ signals (green) in hippocampal neuron processes loaded with FM 4–64 (red). Arrows show increased SPLICS_s_ signals closely associated with active synapses identified by loss of FM 4–64 signal. Time in seconds (s) are shown. Field stimulation was applied at 30s. (MOV 2534 kb)

## Abbreviations

ALS: Amyotrophic lateral sclerosis
ANOVA: Analysis of variance
DIV: Days in vitro
ER: Endoplasmic reticulum
FTD: Frontotemporal dementia
GAPDH: Glyceraldehyde 3-phosphate dehydrogenase
IP3: Inositol 1,4,5-trisphosphate
MAM: Mitochondria associated ER membranes
MAP kinase: Mitogen activated protein kinase
MAP2: Microtubule-associated protein-2
NFH: Neurofilament heavy chain
PBS: Phosphate buffered saline
PDI: Protein disulphide isomerase
PLAs: Proximity ligation assays
PSD95: Post synaptic density protein-95
PTPIP51: Protein tyrosine phosphatase interacting protein-51
s: Seconds
SEM: Standard error of mean
SIM: Structured illumination microscopy
SN: Synaptoneurosomes
SPLICS: Split green fluorescent protein (GFP) ER-mitochondria contact reporter
TBS: Tris-HCl-buffered saline
TOM20: Translocase of the outer mitochondrial membrane protein-20
VAPB: Vesicle-associated membrane protein-associated protein B
